# Risk factors accelerating hypothyroidism in pregnant women referred to health centers in Abadan, Iran

**DOI:** 10.1016/j.dib.2017.07.013

**Published:** 2017-07-14

**Authors:** Mahboobeh Momtazan, Mohammad Javad Mohammadi, Raha Tabahfar, Soraya Rezaee, Aliasghar Valipour, Fatemeh Jamei, Ahmad Reza Yari, Azimeh Karimyan, Sahar Geravandi

**Affiliations:** a Student Research Committee, Abadan School of Medical Sciences, Abadan, Iran; b Abadan School of Medical Sciences, Abadan, Iran; c Research Center for Environmental Pollutants, Qom University of Medical Sciences, Qom, Iran; d School of Public Health, Tehran University of Medical Sciences, Tehran, Iran; e Razi Teaching Hospital, Clinical Research Development Center, Razi Hospital, Ahvaz Jundishapur University of Medical Sciences, Ahvaz, Iran

**Keywords:** Abadan HO, Abadan Health Organization, OCP, Oral Contraceptive Pills, OC, Ovarian Cysts, Hypothyroidism, Pregnant women, Risk factors, Health centers, Iran

## Abstract

The present work contains data obtained during the analysis of pregnant women referred to Abadan Health Centers Organization (Abadan HCO) with confirmed acute hypothyroidism diagnosis. From among all pregnant women referred to Abadan HCO, 600 were chosen consisting of 120 pregnant women from each of the health centers in quintuple areas. In this paper, the effects of family history, occupation, death, abortion, type of diabetes, smoking, lithium consumption, allergy, radiotherapy, ovarian cysts (OC) and oral contraceptive pills (OCP) consumption have been studied (Yassaee et al., 2014) [1]. After completion of the questionnaires by the patients, the obtained coded data were fed into ECSELL software. Statistical analysis of the data was carried out using Special Package for Social Sciences version 16 (SPSS 16).

## Specifications Table

**Table:** 

Subject area	*Medicine, clinical research*
More specific subject area	*Risk factors accelerating hypothyroidism*
Type of data	*Table, figure*
How data was acquired	*Functional clinical assessment of the pregnant women and researcher-made questionnaire analysis*
Data format	*Raw, analyzed, Descriptive and statistical data*
Experimental factors	– *Sample consisted of pregnant women referred to different Abadan Health Centers Organizations.* – *After* Inviting *the pregnant women,* the *researcher-made questionnaire including demographic data as well as the hypothyroidism questionnaires were completed.* – *In this paper, the effects of abortion, smoking, family history, occupation, death, type of diabetes, lithium consumption, allergy, radiotherapy, ovarian cysts (OC) and oral contraceptive pills (OCP) consumption on accelerating hypothyroidism have been studied.*
Experimental features	*Hypothyroidism is one of the factors endangering* pregnant *women.*
Data source location	*Abadan, Iran*
Data accessibility	*Data is included in this article.*

## Value of the data

• These data describe factors affecting acceleration of hypothyroidism in pregnant women and helps with educating the community for the control and prevention of this disease.
• Due to the importance of the risk factors of hypothyroidism in pregnancy (Williams Obstetrics and Gynecology), these factors are discussed in this article.
• The results showed that hypothyroidism can be harmful for pregnant women.
• The results of this study can be used to develop a prevention program to decrease hypothyroidism in pregnant women.
• Results are also important for patients with hypothyroidism especially pregnant women referred to health centers.

## 1 Data

Table 1 represents demographic characteristics of pregnant women referred to Health Centers Organization, Abadan, Iran during 2016 used for description of experiments. Table 2 shows data for factors accelerating hypothyroidism in pregnant women referred to Abadan HCO. Among all factors, family history had the highest score. The results showed that the most important causes of accelerating hypothyroidism in pregnant women were related to the family history (*P*=0.00038). Factors related to hypothyroidism were abortion, smoking, occupation, death, type of diabetes, lithium consumption, allergy, radiotherapy, OC and OCP consumption *P*=0.0035, *P*=0.001, *P*=0.0042, *P*=0.0006, *P*=0.00056, *P*=0.056, *P*=0.0038, *P*=0.06, *P*=0.002, *P*=0.00042, respectively.

**Table 1:** Demographic characteristics of pregnant women referred to health centers.

**Parameter**	**Characteristics**	**Number (in percent)**
Age group	12–19	50 (8.33%)
	20–29	365 (60.83%)
	30–39	150 (25%)
	40–49	30 (5%)
	More than 50	5 (0.84%)
Occupation	Housewife	485 (80.83%)
	Employed	115 (19.17%)
Residence	Urban	600 (100%)
	Rural	0 (0%)
Nationality	Iranian	588 (98%)
	Neighboring Countries (Iraqi, Kuwaiti)	12 (2%)

**Table 2:** Ranking of factors affecting the accelerating hypothyroidism in pregnant women based on their importance.

**Factors**	***P* value**
Family history	0.00038
Occupation	0.0042
Death	0.0006
Abortion	0.0035
Type of diabetes	0.00056
Smoking	0.001
Lithium consumption	0.056
Allergy	0.0038
Radiotherapy	0.06
Ovarian Cysts (Oc)	0.002
Oral Contraceptive Pills (OCP) consumption	0.00042

## 2 Experimental design, materials and methods

### 2.1 Study area description

**Fig. 1:**
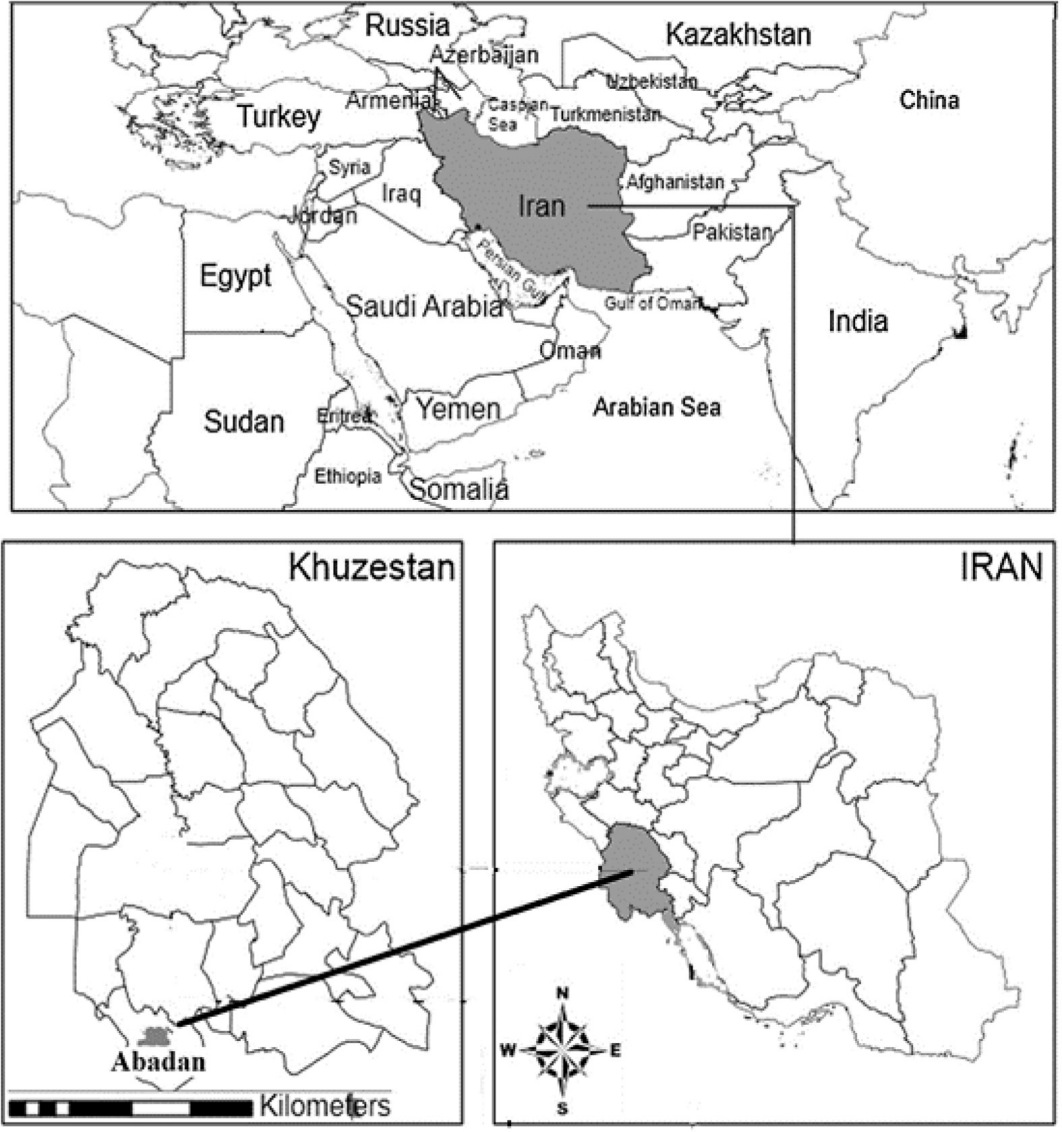
Location of Abadan in the southwest of Iran.

All Health Centers Organizations based in 5 areas (North, South, East, West, and Center) of the city of Abadan were selected. Having a population of 300,000 people, Abadan is one of the metropolitan areas in the Khuzestan province [2]. Khuzestan province climate is hot and semi-humid with long summers and short winters [3], [4],4]. Abadan is Located in the south of Khuzestan province in the southwest of Iran (see Fig. 1).

### 2.2 Experimental design, materials and methods

The five Health Centers Organizations were chosen from around Abadan, Iran. 600 pregnant women referred to Abadan Health Centers Organizations participated in this study. In this study, data was gathered from the thyroid screening program for pregnant women with a gestational age of less than 20 weeks (Including TSH, FT3, FT4, TT3, TT4 tests) as well as a researcher-made questionnaire (based on the risk factors adopted from Williams’ Obstetrics and Gynecology) including the demographic data (e.g. age, sex and experience) and questions which were related to the causes and factors affecting acceleration of hypothyroidism including abortion, smoking, family history, occupation, death, type of diabetes, OC, allergy, radiotherapy, lithium and OCP consumption [5], [6], [7], [8]. Then the collected data were coded and entered into SPSS version 16. Data analysis was performed using SPSS-16. All risk factors were analyzed. The data were analyzed applying descriptive and statistical tests including independent t-test and chi-square.
