# Supplementary material for: Risk factors accelerating hypothyroidism in pregnant women referred to health centers in Abadan, Iran
Source: Data Brief. 2017 Jul 14;14:15–9. doi: 10.1016/j.dib.2017.07.013 (PMC5522916; doi:10.1016/j.dib.2017.07.013)
Supplement: Supplementary file 1 — Supplementary material [file mmc1.doc]

**Risk Factors Accelerating Hypothyroidism in Pregnant Women Referred to Health Centers in Abadan, Iran**

**Conflicts of Interest**

Authors have no conflicts of interest.

**Acknowledgment**

The authors would like to thank Student Research Committee, Abadan School of Medical Sciences for providing financial supported by the grant: (IR.ABADANUMS.REC.1394.8) of this research.

**Funding/Support**

This study was supported by Abadan School of Medical Sciences for providing ethical issue: (IR.ABADANUMS.REC.1394.8) of this research.
